# Circulating Tumor Cells in Prostate Cancer

**DOI:** 10.3390/cancers5041676

**Published:** 2013-12-04

**Authors:** Brian Hu, Holly Rochefort, Amir Goldkorn

**Affiliations:** 1 Institute of Urology, University of Southern California, 1441 Eastlake Avenue, Suite 7416, Los Angeles, CA 90033, USA; E-Mail: brian.hu80@gmail.com; 2 Department of Surgery, University of Southern California, 1520 San Pablo Street, HCT 4300, Los Angeles, CA 90033, USA; E-Mail: Holly.Fish@med.usc.edu; 3 Department of Internal Medicine and Norris Comprehensive Cancer Center, University of Southern California Keck School of Medicine, 1441 Eastlake Avenue, Suite 3440, Los Angeles, CA 90033, USA

**Keywords:** circulating tumor cell, prostate cancer, personalized medicine

## Abstract

Circulating tumor cells (CTCs) can provide a non-invasive, repeatable snapshot of an individual patient’s tumor. In prostate cancer, CTC enumeration has been extensively studied and validated as a prognostic tool and has received FDA clearance for use in monitoring advanced disease. More recently, CTC analysis has been shifting from enumeration to more sophisticated molecular characterization of captured cells, which serve as a “liquid biopsy” of the tumor, reflecting molecular changes in an individual’s malignancy over time. Here we will review the main CTC studies in advanced and localized prostate cancer, highlighting the important gains as well as the challenges posed by various approaches, and their implications for advancing prostate cancer management.

## 1. Introduction

The observation that cancer cells can be found in circulating blood was expounded by Ashworth in 1869 [1]. He drew cancer cells found in the saphenous vein, hoping that this finding would “throw some light upon the mode of origin of multiple tumours”. Since Ashworth’s discovery over 100 years ago, the mechanisms behind cancer metastasis still remain unclear. What has recently improved significantly has been the ability to isolate and study circulating tumor cells (CTCs). These advances have uncovered a field of rich potential, introducing a noninvasive method to accurately monitor and characterize solid malignancies. 

CTCs provide a window into the understanding of metastasis, which can have a profound impact on prostate cancer (PCa). PCa is unique in its high mortality due to metastatic disease combined with a high prevalence of localized disease that does not uniformly lead to death. Incurable and unpredictable metastasis has led to overtreatment of localized disease and undue morbidity. Therefore, better prediction and knowledge of the biology of metastasis can improve care across the spectrum of PCa. 

This paper will examine the biology of CTCs and different modalities for isolation. We will discuss the rationale for their use and review the current literature specific to PCa.

## 2. Biology of CTCs

Metastasis is a multi-step process, involving cellular changes that allow for separation from the primary tumor, intravasation into circulation, survival, and proliferation in a different location. CTCs, cells found in the blood that are shed from the primary tumor or a metastatic deposit, play a key role in the hematogenous spread of a malignancy [2,3,4]. They are rare, with approximately one CTC found per every billion normal cells in a patient with known metastatic cancer [5]. The majority of CTCs are cleared from circulation; however, some can deposit in the bone marrow, termed disseminated tumor cells or seed other sites of metastasis. Interestingly, CTCs can reseed the organ of origin, expressing factors that lead to accelerated tumor growth and angiogenesis [6,7]. CTCs have been found in clusters, potentially representing tumor emboli or a product of intravascular proliferation [8,9]. The exact significance of CTC clusters is unclear, though one study has shown their different gene expression profiles when compared to individual CTCs [10].

Much of the pathology of metastasis is unclear, but emerging data points to epithelial mesenchymal transition (EMT) being involved [11,12]. EMT is the process in which adherent epithelial cells gain migratory and invasive properties. Through this process, cells are able to break through the basement membrane, separate from the tumor, and survive in circulation. The importance of EMT is bolstered by the fact that CTCs demonstrate gains in mesenchymal markers [13]. This process can be reversed, termed mesenchymal epithelial transition, when CTCs may attach and proliferate at a metastatic site [14]. The tumorigenic potential of CTCs has been examined. Carvalho *et al*. isolated CTCs from men with castration-resistant prostate cancer (CRPC) and did not observe any tumor growth when the cells were implanted in mice [15]. This suggests that the majority of human CTCs have little ability to form a tumor using standard xenograft models; there is, therefore, a small subset of aggressive cells that are likely required for metastasis, suggesting heterogeneity of CTC populations. 

## 3. CTC Isolation

**Figure 1:**
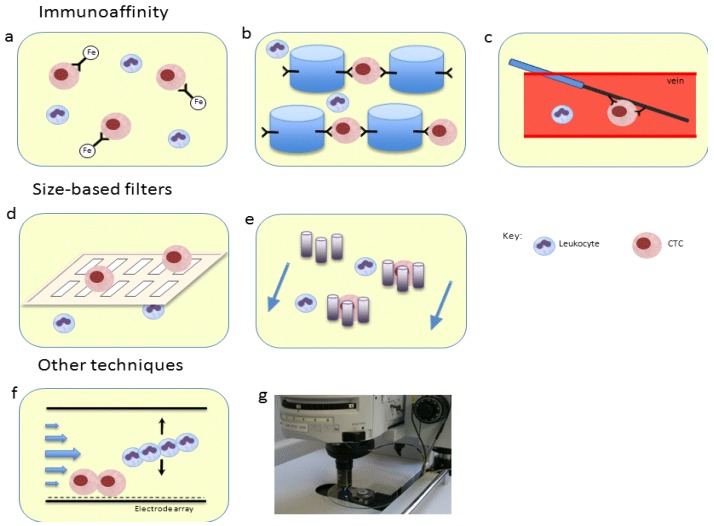
CTC isolation techniques. Immunoaffinity utilizes EpCAM antibodies bound to (**a**) magnetic beads (CellSearch^®^); (**b**) microposts (CTC-chip); or (**c**) an *in-vivo* wire. Size-based techniques use (**d**) pore, slot filters or (**e**) microcrescents. Other techniques include (**f**) dielectrophoresis and (**g**) high speed laser scanning.

The biology and clinical implications of CTCs are highly dependent upon the techniques used to isolate them (Figure 1). Each modality has strengths and limitations in terms of the sensitivity, purity and ability to perform further testing on the cells [16]. Additionally, as there is heterogeneity among CTCs, different enrichment techniques can yield different subpopulations of cells, a fact that impacts further molecular characterization. 

Immunoaffinity is the most common method for isolation, utilizing expression of cell surface markers such as epithelial cell adhesion molecule (EpCAM). The CellSearch^®^ platform (Janssen Diagnostics/Johnson & Johnson, Titusville, NJ, USA) labels magnetic beads with antibodies to EpCAM, drawing out cells with a magnet and staining them with 4',6-diamidino-2-phenylindole (DAPI) nuclear stain and antibodies to cytokeratins and CD 45. The cells are scanned and sent to a reviewer who categorizes CTCs as cells that are nucleated, positive for cytoplasmic cytokeratin, and negative for CD 45. A recent study demonstrated the feasibility of an automated scanning algorithm for CTC detection using CellSearch^®^, an approach which may in time serve as an alternative to human reviewers who can contribute to variability [17]. 

Immunoaffinity techniques are commonly utilized, allow for reliable enrichment of specific subpopulations and have extensive preclinical and clinical data to support their use. CellSearch^®^ is the only method for detecting CTCs in metastatic breast, colon, and PCa that has been cleared by the Food and Drug Administration (FDA) [18]. The major limitation of these techniques is their reliance on cell surface markers, which can have variable or no expression in some malignancies. EpCAM expression, for example, has been shown to be reduced in 29% of prostate cancer samples [19]. This limitation is more pronounced in cells that have undergone EMT, which can allow CTCs to escape detection [20].

CTC-chip is an immunaffinity technique with improved yield of CTCs. Utilizing a microfluidic platform, blood flows through a chip with 78,000 microposts positioned to maximize exposure to cells while minimizing shear forces on the cells [21]. Using CTC-chip with posts coated with antibodies to EpCAM, CTCs were identified in over 99% of patients with various metastatic cancers, including prostate. The CTC-chip proved sensitive in PCa, identifying CTCs in seven out of seven men with localized disease. 

Size-based filters do not rely on cell surface markers but rather on the fact that CTCs are typically larger than other blood cells. Morphometric analyses have demonstrated a spectrum of CTC size depending on the cell line of origin with breast and prostate CTCs typically smaller than CTCs from cervical and liver cancer [22]. All CTCs, however, were significantly larger than white blood cells. Described techniques include filtration through pores, slots or microcrescents [23,24]. These techniques capture a broader spectrum of cells irrespective of cell surface markers and the cells are not bound by antibodies, helping with further analysis. On the other hand, size-based enrichment has potential drawbacks. CTCs may escape capture due to smaller size; conversely, cells that are captured still require subsequent “positive identification” using various immunofluorescent stains (e.g., cytokeratins).

There are a multitude of other techniques to isolate CTCs. Dielectrophoresis separates cells through an electromagnetic method, taking advantage of cells having different polarizable properties [25]. It has been combined with immunoaffinity assays to work synergistically [26]. High speed laser scanning identifies CTCs after staining and is based upon a cell’s morphologic and fluorescence patterns [27]. Another technology, termed NanoVelcro-Chip, utilizes EpCAM antibody-coated silicon wires and has been shown to be easily replicated between different facilities [28]. The addition of laser capture microdissection to this platform allows for pure CTC isolation and sequencing [29]. Still another group utilized a three dimensional tumor cell culture on a microfluidic platform has been shown to successfully culture prostate cancer cells spiked into blood [30]. Lastly, an *in vivo* EpCAM antibody-coated wire placed in a patient’s vein for 30 minutes has shown promising results with regard to CTC capture in breast and lung cancer [31]. 

## 4. Rationale for CTCs in Prostate Cancer

CTC analysis, in many ways, is ideally suited to PCa. To date, CTCs have been most extensively studied and qualified in advanced disease. In castration resistant PCa (CRPC), PSA lacks accuracy in reflecting disease burden and hence is not used as a valid endpoint for clinical trials [32]. Therefore, clinicians are often left with symptoms, imaging or even bone biopsies to define progression or response to treatment. CTC analysis can fulfill the role of a biomarker by serving as an accurate, noninvasive index of disease that can be followed at multiple time points. As a reliable marker, CTCs can be used in clinical trials as an intermediate endpoint, helping expedite the testing of future therapeutics [33]. 

Another potential application for CTCs in PCa, though not as developed, may be their use to differentiate true localized PCa from occult disseminated disease. Imaging studies lack accuracy in this setting and nomograms utilizing clinicopathologic factors are far from definitive [34]. Currently, the most reliable method for determining micrometastases is with surgical lymphadenectomy which may not necessarily reflect hematogenous disease [35]. CTCs can potentially identify patients with early systemic disease, allowing these patients more aggressive, upfront treatment. 

Perhaps most importantly, CTC analysis is rapidly shifting away from simple enumeration and towards molecular characterization. PCa is a heterogeneous disease with different molecular drivers. Selection of treatment for CRPC, however, is largely empiric. CTC analysis (*i.e.*, protein and mRNA expression, DNA mutations or translocations) can characterize a patient’s cancer at different points and in response to different treatments. This may eventually allow clinicians to predict a patient’s response or lack of response to different therapies based on a CTC molecular signature. 

## 5. CTCs in Advanced Prostate Cancer

In an early study of men with metastatic PCa, CellSearch^®^ detected ≥2 CTCs per 7.5 mL blood in 57% of men [36]. In patients with CRPC, >2 CTCs were found in 75% of men with the CellSearch platform [37]. Higher CTC counts were seen in patients with bone metastases and those who had prior chemotherapy, a finding corroborated by Scher *et al*. who demonstrated 81% of men with CRPC who had prior docetaxel chemotherapy had evaluable CTCs during a phase 3 study of abiraterone acetate (AA) [38]. 

CTCs were initially studied in CRPC, where enumeration emerged as an important prognostic tool. Danila *et al*. examined CTC number in relation to survival and found that a higher baseline CTC count was significantly associated with worse survival [37]. De Bono *et al*. studied CTC counts in 231 men with CRPC before and after chemotherapy and found that men with baseline >5 CTCs per 7.5 mL blood had a significantly shorter overall survival (OS) (11.5 *vs*. 21.7 months, *p* < 0.0001) compared to men with ≤5 CTCs [18]. Changes in a patient’s CTC count after treatment were also associated with survival. Patients who experienced a change from favorable (≤5) to unfavorable (>5) CTC count had a significantly worse survival when compared to those with continuously favorable CTC counts (>26 *vs*. 9.3 months, *p* < 0.0001). Conversely, those patients who had a CTC count that decreased with treatment to ≤5 had an almost 15 month improvement in OS (21.3 *vs*. 6.8 months, *p* < 0.0001) compared to those with continuously unfavorable CTC counts. Additionally, CTC count was superior to PSA in predicting survival. These findings ultimately lead to the FDA clearing CellSearch^®^ for evaluation of metastatic CRPC.

**Figure 2:**
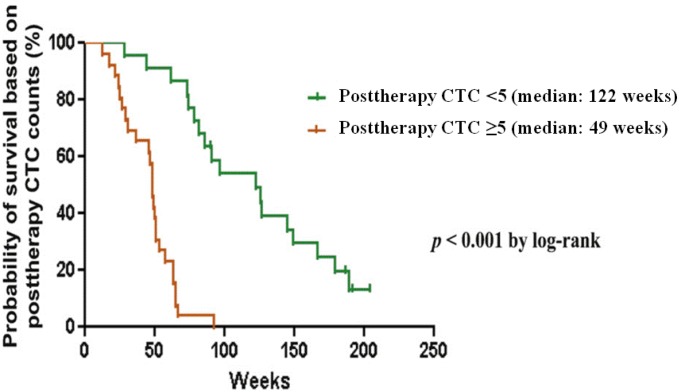
CTC count and survival in castration-resistant prostate cancer. Probability of survival stratified by post-treatment CTC count. CTC count ≥5 at 4 weeks was associated with shorter overall survival compared with patients with CTC counts of <5 (reprinted from [39] with permission of Elsevier).

More recent studies have examined survival stratified by CTC count in patients with CRPC in clinical trials. Danila *et al*. examined survival after treatment with AA during a phase 2 trial, finding a statistically significant decrease in survival if post-treatment CTC count was ≥5 [39] (Figure 2). The median OS was 122 weeks in those with favorable CTC counts compared to 49 weeks in patients with ≥5 CTCs. Scher *et al*. also reported on CTCs obtained in 972 men during a phase 3 trial of AA [38]. CTC count at baseline, changes in CTC count with treatment, and LDH were predictors of OS. The prognostic changes in CTCs were seen as early as 4 weeks after treatment. Recently, our group studied CTCs in a phase 3 trial examining docetaxel with or without atrasentan (SWOG S0421) [40]. CTCs were evaulated in 238 patients both at baseline and 21 days, with the study corroborating several categorical CTC prognostic cutpoints for OS in the prospective, first-line chemotherapy setting [41]. 

**Figure 3:**
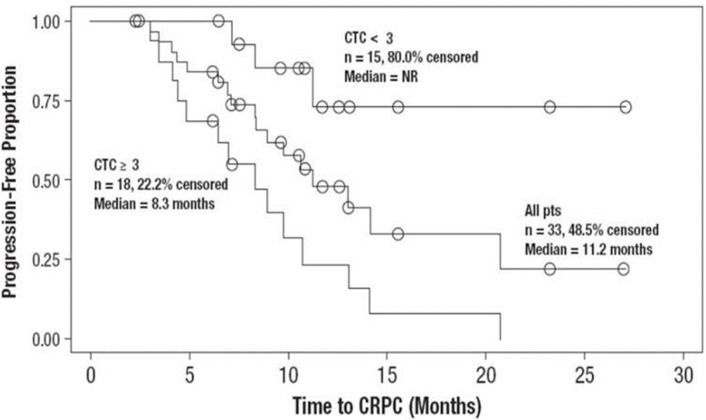
CTC count and time to castration-resistant prostate cancer. Kaplan-Meier survival curves in HSPC (reprinted from [42] with permission of Elsevier).

In hormone-sensitive prostate cancer (HSPC), a few smaller studies have suggested that CTCs may also be prognostic. Goodman *et al*. examined CTCs in 33 patients with HSPC on androgen deprivation therapy, identifying a CTC threshold of 3 cells per 7.5 mL whole blood as a predictor of progression to CRPC (Figure 3) [42]. This group also found CTC count to be an independent predictor of progression to CRPC on multivariable analysis, predicting duration and magnitude of response to androgen deprivation therapy. Resel Folkersma *et al*. also studied CTCs in HSPC, finding CTC count ≥4 was associated with a shorter OS (24 *vs*. 45 months) and PFS (7 *vs*. 44 months) [43]. Amato *et al*. confirmed the associated between survival and ≥5 CTCs across a more heterogeneous population of men with advanced PCa, including those without overt metastatic HSPC [44]. 

**Figure 4:**
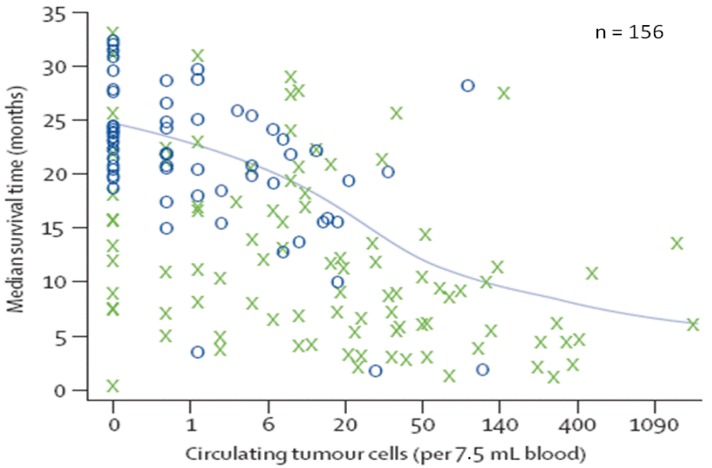
CTC count analyzed as continuous variable. Estimated median survival time according to baseline CTC count. Higher CTC count is associated with worse survival. X represents death; O represents lost to follow-up (reprinted from [45] with permission of Elsevier).

Discrete CTC cutoffs may not be the best reflection of clinical response. Scher *et al*. posited that a decrease from 100 to 10 CTCs may perhaps have more clinical significance than a decrease across a categorical cutpoint of 5, for example from six to four CTCs [45]. To investigate this question, their group analyzed CTC counts as a continuous variable in men with CRPC undergoing chemotherapy (Figure 4). They found that CTC count as a continuous variable, in addition to LDH, was predictive of survival. In the S0421 study, our own CTC correlative studies reaffirmed the use of CTC counts as a continuous variable in men with CRPC [40]. 

CTC enumeration continues to be incorporated into clinical trials in PCa as a secondary endpoint [33]. This includes trials in CRPC testing therapuetics such as AA, cabazitaxel, and TAK-700. One recently published phase 2 trial of temsirolimus in CRPC utilizing change in CTC count as a primary endpoint [46]. 

## 6. CTCs in Localized Prostate Cancer

As would be expected, CTCs appear to be more rare in localized PCa compared with metastatic disease. Early studies on localized PCa with CellSearch^®^ have shown both low yield and limited predictive value of CTCs. Davis *et al*. examined CTCs in localized PCa in 97 men prior to radical prostatectomy, comparing them to 25 men with an elevated PSA but a negative prostate biopsy [47]. Using 30 mL of whole blood, CTCs were found in 21% of patients with PCa and 20% of controls (*p* = 0.946). Another study found no difference in CTC counts between those with localized PCa and healthy controls utilizing the CellSearch^®^ platform [48].

The CTC-chip platform has been used to determine if CTCs are detectable in this setting. Stott *et al*. analyzed CTCs in patients with known PCa (n = 55) and in healthy controls (n = 17) [49]. They isolated CTCs in 8 of the control patients, up to a maximum CTC count of 10. Therefore, they defined ≥14 CTCs as “detectable.” Using this criteria, 8/19 patients (42%) with localized PCa had detectable CTCs. Six of the eight patients with detectable CTCs preoperatively experienced a rapid decline in count within 24 h of surgery, indicating a short half-life. The other two patients had CTCs that declined within 3 months. Of all 19 patients with localized PCa, six had transient increases in CTC values postoperatively, but the clinical significance of these detectable postoperative CTCs in that cohort was not clear. 

Others have utilized RT-PCR for prostate-specific genes from peripheral whole blood as a surrogate for measuring CTCs. Yates *et al*. demonstrated that the presence of perioperative PSA and prostate-specific membrane antigen (PSMA) mRNA improves nomogram prediction of PCa recurrence after radical prostatectomy [50]. Eschwège *et al*. prospectively compared recurrence free survival between patients with detectable (n = 57) and undetectable (n = 53) PSA and PSMA genes in patients undergoing radical prostatectomy. Those with undetectable preoperative mRNA of these prostate-specific genes had a significantly better recurrence free survival (69.6 *vs*. 36.2 months, *p* < 0.0001).

Giesing *et al*. examined antioxidant genes from CTC clusters with a mesh filtration platform in patients with an elevated PSA [51]. They found mRNA levels of three antioxidant genes (GPX1, SOD2, TXNRD1) correlated with the detection of primary PCa. Additionally, continued overexpression of these antioxidant genes predicted disease recurrence after radical prostatectomy. 

CTC count after salvage radiotherapy for localized PCa was studied by Lowes *et al*. [52]. CTCs were detectable in 19/26 patients (73%) prior to radiotherapy. Radiotherapy did not significantly reduce the CTC counts. Patients with good biochemical response were more likely to have decreases in CTC count compared to non-responders, though this did not reach statistical significance. 

Interestingly, there is evidence that even in the setting of localized PCa, CTCs can deposit in the bone marrow, sometimes referred to as disseminated tumor cells (DTCs) [53]. Berg *et al*. evaluated bone marrow aspirates from 272 patients prior to definitive radiotherapy [54]. DTCs were found in 18% of patients and their presence was associated with worse Gleason score and an increased risk of distant metastasis (28% *vs*. 9%, *p* = 0.03). A follow-up study by Lilleby *et al*. identified DTCs in 14% of patients prior to radiotherapy and in 19% of patients after radiotherapy for cT1-3N0M0 disease [55]. The presence of these pretreatment DTCs was the only independent predictor of cancer-specific survival and OS on multivariable analysis. Kollerman *et al*. found that the presence of DTCs in patients treated with neoadjuvant androgen deprivation prior to radical prostatectomy was an independent marker for biochemical relapse [56]. Collectively, these studies suggest that DTCs may, in some cases, constitute occult micrometastatic deposits which may clinically manifest as recurrent disease, *i.e.*, failure of definitive local therapy. 

Though not as robust as the data in advanced PCa, the evidence that CTCs have a role in localized disease continues to accumulate. CTCs may prove useful in differentiating the truly aggressive forms of localized PCa at high risk for recurrence, allowing for multimodal treatment in those select patients. 

## 7. CTCs to Characterize Prostate Cancer

Perhaps more promising than enumeration is the ability to study CTCs in order to molecularly characterize a cancer. The ability to take a “liquid biopsy” at different time points in treatment creates opportunities for therapeutic decisions informed by the specific phenotype of a patient’s cancer, moving closer towards the goal of personalized cancer therapy. Molecular characterization of CTCs, however, represents new challenges. For example, whereas white blood cell contamination may be tolerable when detecting (“yes” or “no”) the presence of disease-related genetic aberrations (e.g., TMPRSS2-ERG fusion product), moving towards more quantitative analyses (e.g., mRNA transcript levels of gene expression) necessitates ultra-pure CTC samples. Technological improvements have helped overcome some of these obstacles, leading to early successes in genomic and transcriptomic profiling of CTCs, sometimes with as little as one cell [57,58,59,60]. 

Our group demonstrated that targeted next generation sequencing of CTCs is possible, sometimes in patients with little evidence of clinical disease (PSA values <1 ng/mL) [61]. This sequencing was able to detect single nucleotide variants (SNVs) in men with HSPC. SNVs have already been shown to be associated with changes in PCa clinical outcomes [62]. Additional profiling was shown to be feasible by Shaffer *et al*., who examined epidermal growth factor receptor expression, chromosome ploidy, and androgen receptor (AR) gene amplification in CTCs [63]. 

Giesing *et al*. performed molecular phenotyping of PCa CTCs with direct links to clinical outcomes [10]. They tested functional gene targets as biomarkers for disease, analyzing 23 targets and identifying overexpression of five genes (SOD2, GPX1, AR, cyclin B and bFGF) that predicted clinical metastases. Three of these genes were able to predict bone metastases independent of pathologic or treatment-related variables. 

Given the important role of EMT in CTCs and metastasis, Chen *et al*. examined EMT-specific genetic profiles [64]. They analyzed CTCs from eight patients with advanced PCa with real time PCR for 84 EMT-related and reference genes. They identified a heterogeneous pattern of expression of EMT-related genes and found that CTCs frequently lost epithelial characteristics. A subset of the EMT-related genes was more frequently expressed in CRPC compared to HSPC.

The TMPRSS2-ERG gene fusion has been implicated in prostate carcinogenesis and castration-resistant growth through disruption of AR signaling and promoting cellular de-differentiation [65]. Attard *et al*. examined expression of the ERG oncogene in patients with CRPC in a clinical trial for AA [66]. They found that ERG status was correlated with PSA decline after treatment with AA. They also found that CTC ERG overexpression persisted in CRPC, with the expression status mirroring that of the original prostate biopsy. This data suggests that the most commonly found tumor on transrectal biopsy is the one that results in hematogenous metastases. This is a notable finding since PCa can be multifocal with clonal heterogeneity and differences in ERG gene expression [67,68]. The importance of ERG was further studies by Danila *et al*. to determine if the TMPRSS2-ERG gene fusion could be used as a marker for sensitivity to AA [39]. In men with CRPC, the gene fusion was found in 15 of 41 (37%) CTC samples. Its presence, however, did not predict PSA decline or response to treatment. 

AR and its signaling axis have been examined in CTCs. Miyamoto *et al*. utilized single cell immunofluorescence to determine the degree of AR signaling within a CTC [69]. They found that first-line androgen deprivation therapy resulted in a transition from an AR-on to AR-off signature. Second-line hormonal therapy in CRPC resulted in more varied changes to the AR signatures. Jiang *et al*. examined mutations in AR, an established escape mechanism to CRPC [70]. They obtained CTCs in men with CRPC, amplified AR by PCR, and detected mutations in 57% of men. Leversha *et al*. utilized fluorescence *in-situ* hybridization in the CTCs of 77 men with CRPC, finding AR gene amplification, another mechanism for castration-resistant growth, in 38% of men [71]. These types of molecular characterization of AR may soon lead to alterations in treatments based upon an individual’s unique AR signature. 

In addition to genetic analyses, other molecular characterizations have been performed, such as quantification of telomerase activity. Activation of telomerase, the enzyme that lengthens telomeres to protect chromosomes, is an important cancer marker in >90% of malignancies [72]. We demonstrated the feasibility of measuring telomerase activity in CTCs, which can act as a functional cancer cell assay across various malignancies [23]. We transitioned this approach to a large PCa phase 3 clinical trial, and showed for the first time in a large prospective setting that a CTC-derived biomarker (telomerase) could be prognostic of OS in a significant subset of men [40,41]. 

## 8. Conclusions

The role of CTCs in PCa is rapidly evolving. CTCs provide a window into the hematogenous spread of cancer and can drastically improve oncologic understanding and patient care. In metastatic PCa, CTC enumeration is an accurate method for monitoring disease and has been used in clinical trials as an intermediate endpoint. CTCs can be detected in localized disease and hold the potential to detect early metastasis. Perhaps the most exciting feature of CTCs is that they provide a platform for noninvasive, repeated inquiries into a cancer’s molecular behavior, ultimately enabling individualized, adaptive and more effective management of PCa over time. 
